# Duration of Menopause, Rather than Primary Hyperparathyroidism Severity, Predicts Osteoporosis in Postmenopausal Women: A Pilot Study from a Spanish Reference Center

**DOI:** 10.3390/jcm14207398

**Published:** 2025-10-20

**Authors:** Ainhoa Arana, Iratxe Ocerin, José I. López, Enrique Echevarría, Gorka Larrinaga

**Affiliations:** 1 Department of Surgery, Cruces University Hospital, 48903 Barakaldo, Spain; 2 Department of Obstetrics and Gynecology, Mendaro Hospital, 20850 Mendaro, Spain; 3 Biobizkaia Health Research Institute, 48903 Barakaldo, Spain; 4 Department of Physiology, Faculty of Medicine and Nursing, University of the Basque Country (UPV/EHU), B. Sarriena, s/n, 48940 Leioa, Spain; 5 Department of Nursing, Faculty of Medicine and Nursing, University of the Basque Country (UPV/EHU), B. Sarriena, s/n, 48940 Leioa, Spain

**Keywords:** osteoporosis, primary hyperparathyroidism, menopause, parathyroidectomy, observational cohort study

## Abstract

**Background/Objectives:** Osteoporosis is considered a surgical indication in primary hyperparathyroidism (PHPT), regardless of menopausal status. This pilot study aimed to evaluate the impact of menopause and PHPT on bone mineral density (BMD) and to explore additional clinical factors that may influence bone health. **Methods:** We conducted an observational pilot study including 204 postmenopausal women with osteopenia or osteoporosis who underwent surgery for sporadic PHPT between 2009 and 2021 at Cruces University Hospital (Spain). Demographic data, anthropometric data, date of last menstrual period, years since menopause (YSM), and the clinical history of bone fragility were collected prior to parathyroidectomy. Biochemical parameters and months with hypercalcemia (MHCa)—as a surrogate for PHPT exposure—were analyzed. BMD results were expressed as a densitometric index, the T-Score. **Results:** Higher age (*p* = 0.043), greater body mass index (BMI) (*p* = 0.039), more YSM (*p* = 0.027), lower serum calcium levels (s-Ca) (*p* = 0.04), and glucocorticoid treatment antecedents (GcT) (*p* = 0.029) were all significantly associated with femoral osteoporosis. Similarly, higher weight (*p* = 0.004), greater MHCa (*p* = 0.01), lower height (*p* = 0.01) and s-Ca levels (*p* = 0.002) were significantly associated with spinal osteoporosis. Furthermore, logistic regression multivariate analysis determined that femur density was independently influenced by YSM (*p* < 0.001), s-Ca (*p* = 0.018), BMI (*p* = 0.002) and GcT (*p* = 0.006). Osteoporosis of the spine was also independently associated with YSM (*p* = 0.036), s-Ca (*p* = 0.031) and also with body weight (*p* = 0.003). **Conclusions:** The duration of menopause (YSM), rather than PHPT severity, is an independent predictor of osteoporosis in postmenopausal women.

## 1. Introduction

Osteoporosis is a disease characterized by a decrease in bone density and alteration of bone microarchitecture, leading to an increased risk of fractures [1]. It is associated with a decline in quality of life, comorbidities, and mortality, making it a public health issue.

Dual-energy X-ray absorptiometry (DEXA) is a standard tool in daily clinical practice and the gold standard for diagnosing osteoporosis [1] through the measurement of bone mineral density (BMD). The international reference, established by the World Health Organization (WHO) [1,2], for diagnosing osteoporosis during menopause is a T-score of −2.5 or lower in the lumbar spine, total hip, or femoral neck [3].

Primary osteoporosis, or age-associated osteoporosis, predominantly occurs in postmenopausal women [4]. Secondary osteoporosis, on the other hand, is associated with medication use, lifestyle habits, or medical conditions [5,6] that have a deleterious effect on bone. Approximately one-third of postmenopausal women also suffer from secondary osteoporosis [4], with primary hyperparathyroidism (PHPT) being a relatively common cause; PHPT predominantly affects women in the first decade after menopause [7,8,9].

Up to 80% of patients with PHPT, characterized by hypercalcemia and elevated or inappropriately high levels of parathyroid hormone (PTH) relative to circulating calcium levels, are asymptomatic [9]. In postmenopausal women around the sixth decade of life, diagnosis is established after finding hypercalcemia in routine blood tests [1] during the evaluation of decreased bone density or osteoporosis. Estrogen deficiency reduces the inhibitory effect of PTH-induced bone resorption, leading to hypercalcemia [7].

Excessive PTH-mediated stimulation leads to varying degrees of hypercalcemia, primarily associated with increased bone resorption and enhanced renal calcium reabsorption. High levels of calcitriol observed in PHPT patients are associated with increased levels of markers of bone remodeling and negatively impact bone mineral density [10]. Changes in bone metabolism in the course of PHPT are associated with the continuous effect of PTH stimulation on bone tissue, leading to increased bone turnover and a predominance of bone resorption over formation. Excess PTH has a predominantly catabolic effect on cortical bone, so patients with PHPT exhibit decreased BMD in cortical bone with relative preservation of trabecular bone mass [11,12,13]. However, in postmenopausal women with PHPT, estrogen deficiency induces early-stage bone loss in trabecular bone (such as in the lumbar spine) [9] and a subsequent increased risk of vertebral fractures [14], even if they are asymptomatic [7]. In postmenopausal women with PHPT, DEXA may therefore underestimate the risk of developing vertebral fractures [13,15], as BMD in the spine may be preserved [13,14]. Consequently, several studies and clinical guidelines since 2014 [16] recommend an additional tool to DEXA that evaluates bone microstructure. Trabecular Bone Score (TBS) [13,17] can identify those postmenopausal women with PHPT who are susceptible to vertebral fractures, regardless of BMD measured by DEXA. However, by the latest international consensus [18], DEXA remains the gold standard, given the disparate results from reviewed studies on the use of TBS in vertebral fractures [19,20].

In 1990, an international group of experts began developing and revising the clinical guidelines for surgical recommendation in asymptomatic PHPT and have continued to do so since then, maintaining the presence of osteoporosis at any location as a criterion for surgical indication [18]. However, despite the higher prevalence of PHPT in postmenopausal women, estrogen deficiency and its effect on bone are not considered.

In the literature, most studies comparing cohorts with surgically treated PHPT to cohorts with untreated PHPT are heterogeneous observational studies; they also disregard menopausal status or other causes of osteoporosis as inclusion criteria [21,22,23]. They describe cohorts as a “low-risk population”, which does not represent the population seen daily in clinical practice.

Some evidence [12] and our clinical experience suggest that the main factor in bone impairment in postmenopausal women with PHPT is the prolonged absence of the protective action of estrogens, rather than the effects of excess PTH. To test this hypothesis, in the present study, we determined the impact of menopause and PHPT on BMD as determined by DEXA in a cohort of surgically treated postmenopausal patients with PHPT (following international clinical guidelines). We also assessed the role of other clinical and pharmacological factors that may coexist in the study population.

## 2. Materials and Methods

### 2.1. Patients

Patients were selected based on these inclusion and exclusion criteria:

**Inclusion Criteria:**

- Caucasian menopausal female patients. Menopause is defined as occurring at 45 years of age.
- Biochemical diagnosis of PHPT characterised by elevated serum calcium levels accompanied by high i-PTH levels (>75 pg/mL) or “inappropriate” normal levels, confirmed in at least two measurements following treatment for 25-hydroxy vitamin D deficiency (optimal range 30–100 ng/mL).
- Patients eligible for surgical treatment: symptomatic PHPT or asymptomatic PHPT meeting the criteria for surgical intervention as determined by international consensus guidelines (Serum calcium >1 mg/dL (0.25 mmol/L) above upper limit of normal. Skeletal features: Fracture by VFA or vertebral X-ray/BMD by T-score≤ −2.5 at any site. Renal features: eGFR or CrCl <60 cc/minute/Nephrocalcinosis or nephrolithiasis on X-ray, ultrasound, or other imaging modality/Urinary calcium excretion >400 mg/day. Age < 50) [16].
- Patients who have provided informed consent for the intervention and potential use of their information for educational and/or research purposes.
- Histologically confirmed diagnosis.
- Bone involvement at diagnosis determined by T-Score [1] on DEXA scan of the femur and/or spine: Low bone density: T-Score between −1 and −2.5 SD. Osteoporosis: T-Score less than −2.5 SD.

**Exclusion Criteria:**

- Patients with PHPT with multiglandular disease.
- Patients with PHPT associated with a known genetic syndrome.
- Patients with parathyroid carcinoma.
- Patients with secondary hyperparathyroidism and tertiary hyperparathyroidism.
- Recurrence of PHPT in patients previously treated with surgery.
- Hypercalcemia due to lithium or thiazide use.
- Previous hormone replacement therapy.

PHPT was determined to be present when the patient had hypercalcemia (normal range 8.1–10.4 mg/dL) and inappropriate or elevated levels of intact PTH (i-PTH), a direct measure of parathyroid gland function independent of renal function (normal range 10–75 pg/mL), in at least two determinations and after treatment of 25-Hydroxy Vitamin D deficiency (optimal range 30–100 ng/mL). Only cases where histological studies confirmed that the tissue was parathyroid, and its weight was pathological (normal range 40–60 mg) [24] were included.

Patients with PHPT associated with multiglandular syndromes, genetic syndromes, parathyroid carcinoma, secondary and tertiary hyperparathyroidism, recurrent PHPT, and lithium- or thiazide-associated hypercalcemia were excluded from the study. To determine the impact of menopause on BMD reduction, patients undergoing hormone replacement therapy were excluded.

According to WHO criteria [25], natural menopause is confirmed after 12 consecutive months without menstruation, provided that the cessation was not due to any other obvious physiological or pathological cause or clinical intervention. The age of onset is specified at 45 years [26], with a range of 45 to 55 years [27]. International clinical guidelines [28] state that laboratory or imaging tests should not be used to diagnose perimenopause or menopause in women aged 45 years or older. Serum FSH measurement should only be considered to confirm menopause in women aged 40–45 with menopause-related symptoms, including changes in menstrual cycle, or in women under 40 in whom premature menopause is suspected. Since all patients in our study had experienced menopause at an age of at least 45 years to meet the inclusion criteria, hormonal testing was not performed, in accordance with the recommendations of the clinical guidelines.

For statistical analysis, women who met the age criterion of 45 years for the onset of menopause and had medically or surgically induced menopause were also considered.

All patients who met the diagnosis of sporadic PHPT with surgical indication [16] and bone involvement in the femur and/or lumbar spine were selected for the study.

### 2.2. Clinical, Laboratory and BMD Evaluation

Demographic, anthropometric, and clinical data and the clinical history of bone fragility were collected for all patients prior to parathyroidectomy.

To determine the etiological factors of bone involvement in this study population, the medical-surgical and pharmacological histories of each patient were considered. This allowed us to determine major causal agents of deleterious bone effects and their possible interactions. To determine the effect of menopause on the bone, the number of years since menopause (YSM) was recorded for each patient at the time of PHPT surgery; likewise, the number of months with hypercalcemia (MHca) prior to surgery was considered as a measure of the bone effect of PHPT. This MHCa was obtained from serial calcium measurements taken before surgical intervention (at least two measurements per patient).

Serum levels of calcium (normal range 8.1–10.4 mg/dL), creatinine (normal range 0.7–1.30 mg/dL), i-PTH (normal range 10–75 pg/mL), 25-Hydroxy Vitamin D (optimal levels >30 ng/mL), and 24-h urinary calcium were recorded before parathyroidectomy. The techniques and analyzers employed for the determination of biochemical parameters were as follows: calcium, creatinine, and 24-h urinary calcium were measured by molecular absorption spectrophotometry using the Arsenazo III reagent; intact parathyroid hormone (iPTH) was determined by a non-competitive chemiluminescent immunoassay; and 25-hydroxy vitamin D was assessed by a competitive chemiluminescent immunoassay. All assays were performed using the Atellica^®^ CH and Atellica^®^ IM analyzers (Siemens Healthineers, Tarrytown, NY, USA).

All patients in the preoperative study underwent abdominal ultrasound to determine the presence or absence of renal lithiasis. BMD was determined before parathyroidectomy using DEXA in the femoral neck, total hip, and lumbar spine (L1-L4) with a QDR 4500 SL machine (Hologic, Waltham, MA, USA). The precision of the technique has an in vivo coefficient of variation of 1.6% for the femoral head and 1% for the lumbar spine. Results are expressed as a densitometric index, the T-Score, which compares BMD to that of a healthy young adult. Treatment with 25-Hydroxy Vitamin D supplements was not considered an exclusion criterion, but it was considered during data analysis.

### 2.3. Statistical Analysis

Data were analyzed using the IBM-SPSS statistical package (Version 28). The Kolmogorov-Smirnov test was used to check the normality of quantitative variables. In our series, all quantitative variables were non-parametric, so comparisons between two variables were made using the Mann-Whitney U test. The analysis of the possible relationship between different qualitative variables was carried out using contingency tables, through Pearson’s chi-squared test.

Mathematical prediction models (multivariate analysis) based on binary logistic regression were used to determine the degree of influence of different independent variables on the dependent variable, osteoporosis of the femur and spine.

In all cases, a *p*-value of < 0.05 was considered significant.

## 3. Results

204 postmenopausal patients with PHPT who met the inclusion criteria were included in the study. Demographic, anthropometric, clinical data and the clinical history of bone fragility are presented in Table 1.

The mean age of the patients was 64.5 ± 8.75 years, with a body mass index (BMI) of 27.3 ± 5.03 kg/m^2^. Among them, 18.1% were smokers.

At the time of PHPT diagnosis, 27% of the patients were asymptomatic, whereas 16.7% had previously experienced fractures; they had a mean duration of 38.89 ± 38.24 MHca and 14.1 ± 8.93 YSM.

The prevalence of chronic morbidities in the study population included 14.2% patients with diabetes mellitus, 7.9% with hyperthyroidism, 3.9% with inflammatory arthropathy, 3.4% with liver disease, 3.4% with hematological disease, 2% with breast cancer, and 1.5% with malabsorption syndrome. Additionally, 52.9% of the patients had been treated for vitamin D insufficiency, 22.5% were on treatment for osteoporosis, 17.3% were taking proton pump inhibitors, 11.3% were on selective serotonin reuptake inhibitor antidepressants, 3.4% were on glucocorticoid treatment, 3.4% on immunosuppressants, and 1% on thyroxine treatment.

Regarding densitometry results (Table 1), 23% of the patients had osteoporosis in the femoral neck, 26% had normal values, and 51% had osteopenia. In the lumbar spine, 41.2% had osteoporosis, 20.1% had normal values, and 38.7% had osteopenia.

For the different statistical analyses, patients were grouped into two categories: Group 1) patients without osteoporosis, including those with normal densitometry and osteopenia (T-score > −2.5); and Group 2) patients with osteoporosis (T-score ≤ −2.5).

We analyzed the association between these quantitative and qualitative clinical variables and osteoporosis in the femur and lumbar spine. The differences in anthropometric, clinical and biochemical parameters between patients with and without osteoporosis in the femur and in lumbar spine are shown in Table 2 (quantitative variables) and Table 3 (qualitative variables).

Patients with femoral osteoporosis were significantly older at diagnosis and had a longer duration of menopause compared with those without osteoporosis. In contrast, body weight, BMI, and serum calcium levels from the last preoperative analysis were lower in women with femoral osteoporosis than in those without (Table 2). Additionally, 57% of patients in the osteoporosis group had a history of glucocorticoid treatment, compared with 42.9% in the non-osteoporotic group (Table 3).

Regarding trabecular bone (lumbar spine), patients with osteoporosis exhibited higher body weight and BMI compared with those without. Serum calcium levels from the last preoperative analysis were also lower in the osteoporosis group (Table 2). Of particular note is the duration of menopause (YSM) at the time of PHPT diagnosis: while the mean duration was 14.1 years, the range in our cohort extended from less than one year to 36 years. The time elapsed from menopause onset to PHPT diagnosis was longer in both women with femoral osteoporosis and those with lumbar spine osteoporosis (Table 2) compared with women without osteoporosis at these sites.

Based on these findings, a multivariate logistic regression analysis was performed including the variables with statistically significant associations. Femoral bone density was independently influenced by YSM, serum calcium levels, BMI, and prior glucocorticoid treatment (Table 4), whereas lumbar spine bone density was independently associated with YSM, serum calcium levels, and body weight (Table 5).

## 4. Discussion

This study analyzed the influence of different clinical, anthropometric and demographic variables on femur and vertebral column osteoporosis in postmenopausal women with PHPT. The results demonstrated that YSM, BMI, weight and height, glucocorticoid treatment, and serum calcium levels were associated with osteoporosis. Furthermore, multivariate analysis showed that YSM and lower serum calcium are independent variables predicting the onset of osteoporosis in both the femur and spine.

PHPT is an endocrine disorder prevalent in postmenopausal women during the 5th–6th decade of life [7,8,9]. However, studies considering menopausal status as a variable influencing bone health in these patients are scarce. Consequently, the international consensus from 1990 to 2022 [18] on recommendations for surgery in asymptomatic PHPT patients is that YSM is not a potentially significant factor affecting BMD.

Estrogens inhibit osteoclastic activity and bone resorption, making bones more resistant to the effects of PTH [26]. It can therefore be inferred that longer estrogen deprivation due to menopause may reduce protection against the deleterious effects of excessive PTH in PHPT patients [12]. Based on this premise, this study analyzed the influence of menopausal duration on BMD in these patients.

In our series, 41% of patients had osteoporosis in the spine and 23% in the femur. Given that excessive PTH leads to a more pronounced decrease in BMD in cortical bone compared to trabecular bone [12], other variables like menopausal duration may play a significant role in trabecular bone demineralization in these patients.

In our multivariate analysis, we used YSM and MHca as necessary variables to determine the influence of menopause and PHPT on bone, respectively. The MHCa was obtained from serial calcium measurements taken before surgical intervention, comprising at least two measurements per patient. Quantitative variables (height, weight, BMI, and age) and qualitative variables (glucocorticoid treatment) associated with osteoporosis were also included. The multivariate analysis confirmed the study’s hypothesis that YSM is an independent predictive variable for osteoporosis in both the femur and spine. This finding is consistent with Rogmanoli et al. [12], who reported that menopausal duration is more important than chronological age in predicting vertebral fractures in these patients.

Patients with osteoporosis, both in the femur and lumbar spine, exhibited significantly lower serum calcium levels than those without osteoporosis. This finding correlates with current clinical practice, where most diagnoses of PTHT are established in asymptomatic patients during the first decade of menopause through the examination of osteoporosis by serum calcium and i-PTH checks [29]. Furthermore, our osteoporosis group exhibited lower MHCa, i-PTH level, and even weight of the adenoma than the non-osteoporosis group. Routine measurement of serum calcium or i-PTH is not performed in asymptomatic patients without osteoporosis; in this group, therefore, the diagnosis of PTHT is not made at such an early stage. Therefore, this study does not relate the severity of primary hyperparathyroidism, in terms of calcium levels, duration of the condition, and PTH, to the risk of developing osteoporosis. A recent publication by Corbetta et al. [30] describes results in the same direction.

We also observed that patients with higher BMI due to higher body weight had a lower risk of developing osteoporosis in the femur and spine. Several studies support this result [31,32,33] suggesting total body weight and BMI are protective factors against osteoporosis in menopausal women, possibly due to the role of adipose tissue in maintaining circulating estrogens via peripheral androgen aromatization [34]. Patients with osteoporosis in the spine also tended to have shorter height. This may be due to osteoporosis at this level reducing vertebral body height [35].

Finally, this study identified daily glucocorticoid use exceeding 5 mg for more than three months as an independent predictor for femur osteoporosis. However, multivariate analysis did not indicate the same for the lumbar spine. Recent studies indicate that bone densitometry is more reliable in assessing bone microstructure damage in the femur than in the lumbar spine in patients receiving high-dose and/or chronic glucocorticoid treatment [36]. Therefore, TBS is recommended as a complementary tool to standard densitometry in secondary osteoporosis [17], including steroid-induced osteoporosis and that in the spine of menopausal patients with PHPT [12]. However, few clinical centers routinely use this tool.

In conclusion, in line with our hypothesis, our results reveal that postmenopausal women with PHPT and longer estrogen deprivation, as measured by YSM, are at greater risk of developing osteoporosis. Moreover, the influence of this variable on osteoporosis outweighs factors closely related to PHPT, such as duration of hypercalcemia, i-PTH levels, and calcium excretion. Hence, a multidisciplinary clinical approach is recommended for these patients, emphasizing not only surgical indications following current protocols but also prevention, treatment, and monitoring by menopause specialists.

It is important to acknowledge that the main limitation of this pilot study lies in its retrospective design, which prevents full control over potential sources of bias, including selection bias, as patient inclusion was non-random. Furthermore, the exclusion criteria limited the final sample size, which may restrict the generalizability of our findings to broader populations. The study was sufficiently powerful for detecting large effects but likely underpowered with respect to identifying small-to-moderate associations. Therefore, although the study was conducted at a leading university hospital in northern Spain and included a cohort spanning from 2009 to 2021, expanding the sample in future multicenter studies will be essential to validate and consolidate these results.

## Figures and Tables

**Table 1:** **Baseline characteristics of the study population (*n* = 204)**. Continuous variables are reported as the mean and standard deviation, and categorical variables as the percentage of total patients.

Age (years)	64.5 ± 8.75
BMI (kg/m^2^)	27.3 ± 5.03
Smoking habit	18.1%
YSM	14.1 ± 8.93
Surgical menopause	13.2%
MHca (months)	38.9 ± 38.24
Asymptomatic	27.0%
Patients with fractures	16.7%
***Comorbidities***	
Diabetes mellitus	14.2%
Hyperthyroidism	7.9%
Inflammatory arthropathy	3.9%
Liver disease	3.4%
Haematological disease	3.4%
Breast cancer	2.0%
Malabsorptive syndrome	1.5%
***Chronic Treatments***	
P-pi	17.3%
Antidepressants	11.3%
Glucocorticoids	3.4%
Immunosuppressants	3.4%
Thyroxine	1.0%
25-OH-Vitamin D deficiency	52.9%
Osteoporosis	22.5%
***Biochemistry***	
s-Ca (mg/dL)	11.3 ± 0.73
i-PTH (pg/mL)	201.7 ± 203.41
25-OH-Vitamin D (ng/mL)	20.9 ± 10.78
24-h urine calcium (mg/24 h)	338.3 ± 169.47
***Bone mineral density (BMD)***	
Femoral neck (%)	N 26%, OSTP 51%, OSPR 23%
Lumbar spine (%)	N 20%, OSTP 39%, OSPR 41%

BMI: body mass index; YSM: years since menopause; MHca: months with hypercalcemia. P-pi: proton-pump inhibitors. Reference values: s-Ca (serum calcium): 8.1–10.4 mg/dL; i-PTH (intact parathormone): 10–75 pg/mL; 25-OH-Vitamin D: insufficiency < 20 ng/mL; 24-h urine calcium < 300 mg/24 h. BMD: Normality was defined as a T score greater than −1 SD; osteopenia: a T score between −1 and −2.5 SD; osteoporosis: a T score equal to or less than −2.5 SD.

**Table 2:** **Influence of quantitative clinical and analytical variables on femoral and spinal osteoporosis (OSPR)**. Values are expressed as mean ± standard error. Mann-Whitney U test was employed to compare quantitative variables. Significant *p*-values (*p* < 0.05) are highlighted in **bold**.

Quantitative Variables	Without Femoral OSPR **(*n* = 157)**	With Femoral OSPR (*n* = 47)	*p* =	Without Spinal OSPR (*n* = 120)	With Spinal OSPR (*n* = 84)	*p* =
Age at diagnosis (years)	63.81 ± 0.704	66.79 ±1.195	**0.043**	64.14 ± 0.851	65.00 ± 0.863	0.505
Weight (kg)	68.33 ± 1.005	63.60 ± 1.723	**0.032**	69.38 ± 1.170	64.19 ± 1.264	**0.004**
Height (cm)	1.57 ± 0.005	1.56 ± 0.09	0.548	1.58 ± 0.006	1.56 ± 0.006	**0.010**
BMI (kg/m^2^)	27.68 ± 0.403	25.99 ± 0.711	**0.039**	27.88 ± 0.468	26.47 ± 0.530	0.055
Previous pregnancies (*n* =)	2.15 ± 0.109	2.19 ± 0.227	0.800	2.14 ± 0.123	2.19 ± 0.163	0.825
Age at menopause (years)	50.52 ± 0.225	50.02 ± 0.456	0.316	50.49 ± 0.273	50.29 ± 0.300	0.827
YSM	13.29 ± 0.693	16.76 ± 1.355	**0.027**	13.65 ± 0.858	14.71 ± 0.897	0.301
MHca	40.89 ± 3.304	31.93 ± 5.241	0.200	41.25 ± 3.535	35.64 ± 4.634	**0.038**
i-PTH (pg/mL)	206.86 ± 18.186	184.76 ± 11.408	0.396	223.07 ± 23.36	171.34 ± 8.236	0.290
s-Ca (mg/dL)	11.40 ± 0.061	11.15 ± 0.080	**0.040**	11.45 ± 0.065	11.19 ± 0.078	**0.002**
Calcium excretion (mg/24 h)	343.99 ± 13.806	322.11 ± 25.555	0.406	331.78 ± 16.749	348.75 ± 17.322	0.312
25-hydroxyvitaminD (ng/mL)	20.571 ± 0.840	21.98 ± 1.817	0.603	20.003 ± 0.869	22.14 ± 1.385	0.485
Weight of parathyroid adenoma (mg)	1117.71 ± 102.980	746.11 ± 84.852	0.104	1043.98 ± 106.8	1015.11 ± 129.76	0.975

OSPR: Osteoporosis; BMI: body mass index; YSM: years since menopause; MHca: months with hypercalcemia. i-PTH, intact parathyroid hormone; s-Ca, serum calcium.

**Table 3:** **Influence of qualitative clinical and analytical variables on femoral and spinal osteoporosis (OSPR).** Values are expressed as number of patients. Chi-square test was employed to compare qualitative variables. Significant *p*-values (*p* < 0.05) are high-lighted in **bold**.

Qualitative Variables	Without Femoral OSPR (*n* = 157)	With Femoral OSPR (*n* = 47)	*p* =	Without Spinal OSPR (*n* = 120)	With Spinal OSPR (*n* = 84)	*p* =
**Smoking habit** NO YES	146 11	41 6	0.445	113 7	74 10	0.303
**Induced menopause** NO YES	136 21	41 6	0.914	105 15	72 12	0.711
**Diabetes Mellitus** NO YES	132 25	43 4	0.202	102 18	73 11	0.701
**Hyperthyroidism** NO YES	144 13	44 3	0.125	111 9	77 7	0.487
**Previous breast cancer** NO YES	155 2	45 2	0.196	118 2	82 2	0.717
**Prev. glucocorticoid treatment** NO YES	154 3	43 4	**0.029**	116 4	81 3	0.927
**Prev. immunosuppressant treatment** NO YES	152 5	45 2	0.724	114 6	83 1	0.141
**Prev. proton-pump inhibitor treatment** NO YES	126 31	42 5	0.151	101 19	67 17	0.417
**Prev. antidepressant treatment** NO YES	138 19	43 4	0.495	106 14	75 9	0.832
**Prev. Vit D treatment** NO YES	73 84	23 24	0.769	58 62	38 46	0.663

OSPR: Osteoporosis.

**Table 4:** **Predictive (multiple logistic regression) model according to femur osteoporosis at diagnosis of PHPT**.

	Variables	*p* =	B	ExpB	CI (Inf)	CI (Sup)
**Multiple logistic regression**	YSM	0.411	0.058	1.060	0.922	1.218
	MHca	0.209	−0.007	0.993	0.982	1.004
	s-Ca	**0.024**	−0.785	0.456	0.231	0.902
	Age	0.713	0.028	1.028	0.888	1.190
	BMI	**0.002**	−0.146	0.864	0.788	0.948
	GcT	**0.007**	2.410	11.135	1.934	64.106
**Final Step of the Wald method**	**YSM**	**<0.001**	0.081	1.084	1.034	1.137
	**s-Ca**	**0.018**	−0.841	0.431	0.214	0.867
	**BMI**	**0.002**	−0.143	0.867	0.792	0.950
	**GcT**	**0.006**	2.454	11.632	2.047	66.102

Selected independent variables were years since menopause (YSM), months with hypercalcemia (MHca), calcemia (s-Ca), age, body mass index (BMI) and glucocorticoid treatment (GcT). A stepwise selection procedure (backwards Wald method) was used to select the final optimal model. ExpB with confidence interval (CI) is also included. According to the Omnibus test, the model was statistically significant (*p* < 0.001). Hosmer–Lemeshow test (*p* = 0.640). R2 Nagelkerke (*p* = 0.224). Statistically significant *p*-values are highlighted in **bold**.

**Table 5:** **Predictive model (multiple logistic regression model) according to spine osteoporosis at diagnosis of PHPT**.

	Variables	*p* =	B	ExpB	CI (Inf)	CI (Sup)
**Multiple logistic regression**	YSM	0.080	0.034	1.034	0.996	1.074
	MHca	0.217	−0.005	0.995	0.987	1.003
	s-Ca	**0.027**	−0.545	0.580	0.357	0.940
	Weight	**0.010**	−0.037	0.963	0.936	0.991
	Height	0.164	−3.964	0.019	0.000	5.026
**Final Step of the Wald method**	**YSM**	**0.036**	0.039	1.040	1.003	1.078
	**s-Ca**	**0.031**	−0.531	0.588	0.363	0.953
	**Weight**	**0.003**	−0.042	0.959	0.933	0.986

Selected independent variables were years since menopause (YSM), months with hypercalcemia (MHca), serum calcium levels (s-Ca), weight, and height. A stepwise selection procedure (backwards Wald method) was used to select the final optimal model. ExpB with confidence interval (CI) is also included. According to the Omnibus test, the model was statistically significant (*p* = 0.002). Hosmer–Lemeshow test (*p* = 0.746). R2 Nagelkerke (*p* = 0.137). Statistically significant *p*-values are highlighted in **bold**.

## Data Availability

The dataset analyzed during the study are not publicly available due to privacy and confidentiality concerns. However, researchers may apply for data access through the Research Ethics Committee of the University Hospital of Cruces.

## References

[1] McClung M.R., Pinkerton J., Blake J., Cosman F., Lewiecki E., Shapiro M. (2021). Management of osteoporosis in postmenopausal women: The 2021 position statement of The North American Menopause Society. Menopause.

[2] Kanis J.A. (1994). Assessment of fracture risk and its application to screening for postmenopausal osteoporosis: Synopsis of a WHO report. WHO Study Group. Osteoporos. Int..

[3] Kanis J.A., Adachi J.D., Cooper C., Clark P., Cummings S.R., Diaz-Curiel M., Harvey N., Hiligsmann M., Papaioannou A., Pierroz D.D. (2013). Standardising the descriptive epidemiology of osteoporosis: Recommendations from the Epidemiology and Quality of Life Working Group of IOF. Osteoporos. Int..

[4] Shevroja E., Lamy O., Kohlmeier L., Koromani F., Rivadeneira F., Hans D. (2017). Use of Trabecular Bone Score (TBS) as a Complementary Approach to Dual-energy X-ray Absorptiometry (DXA) for Fracture Risk Assessment in Clinical Practice. J. Clin. Densitom..

[5] Cosman F., de Beur S.J., LeBoff M.S., Lewiecki E.M., Tanner B., Randall S., Lindsay R. (2014). Clinician’s Guide to Prevention and Treatment of Osteoporosis. Osteoporos. Int..

[6] Camacho P.M., Petak S.M., Binkley N., Diab D.L., Eldeiry L.S., Farooki A., Harris S.T., Hurley D.L., Kelly J., Lewiecki E.M. (2020). American association of clinical endocrinologists/american college of endocrinology clinical practice guidelines for the diagnosis and treatment of postmenopausal osteoporosis-2020 update. Endocr. Pract..

[7] Rubin M.R., Bilezikian J.P., McMahon D.J., Jacobs T., Shane E., Siris E., Udesky J., Silverberg S.J. (2008). The natural history of primary hyperparathyroidism with or without parathyroid surgery after 15 years. J. Clin. Endocrinol. Metab..

[8] Mazeh H., Sippel R.S., Chen H. (2012). The role of gender in primary hyperparathyroidism: Same disease, different presentation. Ann. Surg. Oncol..

[9] Bilezikian J.P. (2018). Primary Hyperparathyroidism. J. Clin. Endocrinol. Metab..

[10] Iwanowska M., Kochman M., Szatko A., Zgliczyński W., Glinicki P. (2024). Bone Disease in Primary Hyperparathyroidism-Changes Occurring in Bone Metabolism and New Potential Treatment Strategies. Int. J. Mol. Sci..

[11] Vignali E., Viccica G., Diacinti D., Cetani F., Cianferotti L., Ambrogini E., Banti C., Del Fiacco R., Bilezikian J.P., Pinchera A. (2009). Morphometric vertebral fractures in postmenopausal women with primary hyperparathyroidism. J. Clin. Endocrinol. Metab..

[12] Romagnoli E., Cipriani C., Nofroni I., Castro C., Angelozzi M., Scarpiello A., Pepe J., Diacinti D., Piemonte S., Carnevale V. (2013). “Trabecular Bone Score” (TBS): An indirect measure of bone micro-architecture in postmenopausal patients with primary hyperparathyroidism. Bone.

[13] Santos L.M.D., Ohe M.N., Pallone S.G., Nacaguma I.O., Kunii I.S., da Silva R.E.C., Vieira J.G.H., Lazaretti-Castro M. (2021). Trabecular Bone Score (TBS) in Primary Hyperparathyroidism (PHPT): A Useful Tool?. J. Clin. Densitom..

[14] De Geronimo S., Romagnoli E., Diacinti D., D’Erasmo E., Minisola S. (2006). The risk of fractures in postmenopausal women with primary hyperparathyroidism. Eur. J. Endocrinol..

[15] Muñoz-Torres M., Manzanares Córdova R., García-Martín A., Avilés-Pérez M.D., Serrano R.N., Andújar-Vera F., García-Fontana B. (2019). Usefulness of Trabecular Bone Score (TBS) to Identify Bone Fragility in Patients with Primary Hyperparathyroidism. J. Clin. Densitom..

[16] Bilezikian J.P., Brandi M.L., Eastell R., Silverberg S.J., Udelsman R., Marcocci C., Potts J.T. (2014). Guidelines for the management of asymptomatic primary hyperparathyroidism: Summary statement from the Fourth International Workshop. J. Clin. Endocrinol. Metab..

[17] Shevroja E., Reginster J.Y., Lamy O., Al-Daghri N., Chandran M., Demoux-Baiada A.-L., Kohlmeier L., Lecart M.-P., Messina D., Camargos B.M. (2023). Update on the clinical use of trabecular bone score (TBS) in the management of osteoporosis: Results of an expert group meeting organized by the European Society for Clinical and Economic Aspects of Osteoporosis, Osteoarthritis and Musculoskeletal Diseases (ESCEO), and the International Osteoporosis Foundation (IOF) under the auspices of WHO Collaborating Center for Epidemiology of Musculoskeletal Health and Aging. Osteoporos. Int..

[18] Bilezikian J.P., Khan A.A., Silverberg S.J., Fuleihan G.E.-H., Marcocci C., Minisola S., Perrier N., Sitges-Serra A., Thakker R.V., Guyatt G. (2022). Evaluation and Management of Primary Hyperparathyroidism: Summary Statement and Guidelines from the Fifth International Workshop. J. Bone Miner. Res..

[19] Grigorie D., Coles D., Sucaliuc A. (2018). Trabecular bone score (tbs) has a poor discriminative power for vertebral fractures in 153 romanian patients with primary hyperparathyroidism. Acta Endocrinol..

[20] Liu M., Williams J., Kuo J., Lee J.A., Silverberg S.J., Walker M.D. (2019). Risk factors for vertebral fracture in primary hyperparathyroidism. Endocrine.

[21] Ramos L., Piedra M., Muñoz P., Vázquez L.A., García-Unzueta M.T., Montalbán C., Amado J.A. (2019). Bone mineral density evolution and incidence of fractures in a cohort of patients with primary hyperparathyroidism treated with parathyroid surgery vs. active surveillance during 6 years of follow-up. Evolución de la densidad mineral ósea y aparición de fracturas en una cohorte de pacientes con hiperparatiroidismo primario tratados con cirugía paratiroidea vs. vigilancia activa sin cirugía en 6 años de seguimiento. Endocrinol. Diabetes Nutr. (Engl. Ed.)..

[22] Jones A.R., Simons K., Harvey S., Grill V. (2022). Bone Mineral Density Compared to Trabecular Bone Score in Primary Hyperparathyroidism. J. Clin. Med..

[23] Oprea T.E., Barbu C.G., Martin S.C., Sarbu A.E., Duta S.G., Nistor I.M., Fica S. (2023). Degraded Bone Microarchitecture in Women with PHPT-Significant Predictor of Fracture Probability. Clin. Med. Insights Endocrinol. Diabetes..

[24] Gómez-Ramírez J., Arranz Jiménez R. (2024). Surgical tactics of parathyroidectomy: Should we abandon the use of ioPTH?. Am. J. Surg..

[25] World Health Organization Scientific Group (1996). Research on the menopause in the 1990s. Report of a WHO Scientific Group. World Health Organ Tech. Rep. Ser..

[26] Villarroya-Marquina I., Lorente-Poch L., Sancho J., Sitges-Serra A. (2020). Influence of gender and women’s age on the prevalence of parathyroid failure after total thyroidectomy for multinodular goiter. Gland. Surg..

[27] Sowers M.R., Zheng H., McConnell D., Nan B., Harlow S., Randolph J.F. (2008). Follicle stimulating hormone and its rate of change in defining menopause transition stages. J. Clin. Endocrinol. Metab..

[28] (2024). Menopause: Identification and Management; National Institute for Health and Care Excellence (NICE): London, UK. https://pubmed.ncbi.nlm.nih.gov/31940155/.

[29] Minisola S., Arnold A., Belaya Z., Brandi M.L., Clarke B.L., Hannan F.M., Hofbauer L.C., Insogna K.L., Lacroix A., Liberman U. (2022). Epidemiology, Pathophysiology, and Genetics of Primary Hyperparathyroidism. J. Bone Miner. Res..

[30] Corbetta S., Gianotti L., Castellano E., Carrara S., Raineri F., Munari E., Guabello G., Cairoli E., Chiodini I., Giovanelli L. (2024). Skeletal phenotypes in postmenopausal women affected by primary hyperparathyroidism. Front. Endocrinol..

[31] Johansson H., Kanis J.A., Odén A., McCloskey E., Chapurlat R.D., Christiansen C., Cummings S.R., Diez-Perez A., A Eisman J., Fujiwara S. (2014). A meta-analysis of the association of fracture risk and body mass index in women. J. Bone Miner. Res..

[32] Wu S.F., Du X.J. (2016). Body Mass Index May Positively Correlate with Bone Mineral Density of Lumbar Vertebra and Femoral Neck in Postmenopausal Females. Med. Sci. Monit..

[33] Mornar M., Novak A., Bozic J., Vrdoljak J., Kumric M., Vilovic T., Rakovic I., Kurir T.T., Martinovic D., Urlic H. (2024). Quality of life in postmenopausal women with osteoporosis and osteopenia: Associations with bone microarchitecture and nutritional status. Qual. Life Res..

[34] López-Gómez J.J., Pérez Castrillón J.L., de Luis Román D.A. (2016). Impact of obesity on bone metabolism. Influencia de la obesidad sobre el metabolismo óseo. Endocrinol. Nutr..

[35] Pluskiewicz W., Adamczyk P., Werner A., Bach M., Drozdzowska B. (2023). Height Loss Is an Independent Predictor of Fracture Incidence in Postmenopausal Women: The Results from the Gliwice Osteoporosis Study (GO Study). Biomedicines.

[36] Kobza A.O., Herman D., Papaioannou A., Lau A.N., Adachi J.D. (2021). Understanding and Managing Corticosteroid-Induced Osteoporosis. Open Access Rheumatol..

